# Caregivers had neighbourhood support but perceived it unsatisfactory and worsened: England Community Life Survey, 2012–2014

**DOI:** 10.1007/s11356-017-8701-6

**Published:** 2017-03-11

**Authors:** Ivy Shiue

**Affiliations:** 1 Department of Health and Life Sciences, Northumbria University, Newcastle upon Tyne, England NE7 7XA UK; 2 Alzheimer Scotland Dementia Research Centre, University of Edinburgh, Edinburgh, UK

**Keywords:** Neighbourhood epidemiology, Caregiver, Satisfaction, Social support, Built environment, Community support, Mental health

## Abstract

There has been limited research studying neighbourhood support for caregivers. Therefore, the aim of the present study was to investigate the support from neighbourhoods between both caregivers and non-caregivers in a country-wide and population-based setting. Data were retrieved from England Community Life Survey, 2012–2014, a new annual household survey conducted by face-to-face interview since 2012, with a representative sample size of 5–6000 adult (aged 16 years and over) resident per year in England. Chi-square test and logistic regression modelling were performed to examine the variance in support from and perception toward neighbourhoods between caregivers and non-caregivers. Of 15,320 study participants, 2315 (16.0%) had a caring responsibility. There was not much variance in feeling belonging, comfortably asking neighbours to keep keys, comfortably asking neighbours to mind children, believing neighbours pulling together and trusting people in the neighbourhood between caregivers and non-caregivers. However, caregivers seemed to be more likely to chat to neighbours (OR 0.77, 95% CI 0.69–0.87, *P* < 0.001) and comfortably ask neighbours to help collect grocery (OR 0.89, 95% CI 0.81–0.98, *P* = 0.023). In addition, caregivers tended to perceive their neighbourhoods unsatisfactory (OR 1.17, 95% CI 1.05–1.32, *P* = 0.007) and worsened in the last 2 years (OR 1.36, 95% CI 1.22–1.51, *P* < 0.001). For future research, a longitudinal neighbourhood monitoring surveillance for all people would be suggested. For practice and policy, environmental health and nursing programs might need to extend education trainings and interventions to cover all neighbourhoods at different time points that can lessen both disease and caregiving burden and therefore optimize health and quality of life.

## Introduction

### Evidence before this study

Caregiving is a multi-dimensional, long and complex process. The recent two systematic reviews on dementia and stroke caregivers have summarized that patient behavioural problems, caregiver coping and personality traits and competence are the most consistent determinants of caregiver burden, depression and mental health (van der Lee et al. 2014; Rigby et al. 2009). It has been long known that social support, the perception and actuality when one is cared for, in various forms is important for everyone, and it could be particularly critical for caregivers (Vrabec 1997; Clipp and George 1990). It is used to prevent from primary and secondary poor mental health such as depression and functional impairment and to help strengthen the coping with burden of caregiving (Kate et al. 2013; Rodakowski et al. 2012; Demirtepe-Saygili and Bozo 2011; Ownsworth et al. 2010; Upton and Reed 2006; Miller et al. 2001; MaloneBeach and Zarit 1995).

### Environmental factors on caregivers

Caregivers could be exposed to various environmental hazards in the neighbourhood (Kliewer et al. 2004; Ondersma et al. 2006; Proctor et al. 2011). These risks could come in physical, mental or even financial forms that would threaten one’s stability and consequently health concerns and the work performance (Brummett et al. 2005). Therefore, physical and mental supports from neighbourhoods would be important because it is the immediate living surrounding that people have been facing daily.

### Knowledge gap

Neighbourhoods, as part of the built and social environments to support human living, could influence one’s health and wellbeing, being physical or mental (Bender et al. 2015; Christian et al. 2015; Shiue 2015; Barnes et al. 2011: Yen et al. 2009; Diez et al. 2001). Neighbourhood perception, either on the physical surroundings or the settlement feelings, could also act as a mediator between neighbourhoods and human behaviours, health and wellbeing (Tappe et al. 2013; Martin-Storey et al. 2012; Siordia et al. 2012; Gary et al. 2008). One of the dimensions for such perception would be neighbourhood satisfaction. Research has continuously shown how one’s neighbourhood satisfaction could be associated with one’s health conditions and health behaviours (Halbert et al. 2015; Shiue 2014; de Jong et al. 2012; Leslie and Cerin 2008; Morris et al. 2008). However, none of these studies focused on caregivers.

### Study aim

Following this context, therefore, the aim of the present study was to investigate and understand the neighbourhood support among caregivers and their perception toward them in a country-wide and population-based setting.

## Methods

### Study sample

Data were retrieved from the UK Community Life Survey, 2012–2014 (more details via https://www.gov.uk/government/collections/community-life-survey), with two study waves combined in the cross-sectional analysis. It has been a new annual household survey conducted by face-to-face interview since 2012, with a sample size of 5–6000 adult (aged 16 years and over) resident per year in England. The survey tracks the latest trends and developments across areas key to encouraging social action and empowering communities (more details via http://www.commlife.co.uk/). To ensure the results to be representative of residents (random probability sampling; robust, nationally representative data on behaviours and attitudes within communities to inform and direct policy and action in these areas) in England, quality assurance was implemented (more details via https://www.gov.uk/government/publications/faqs-for-the-community-life-survey-and-supporting-documents/faqs-and-information-about-the-community-life-survey).

### Variables and analyses

In the present study, caregivers were defined as those who had a caring responsibility (question, and do you have any caring responsibilities for a member of your immediate family or a close relative outside of your household who has any long-standing illness, disability or infirmity?). Neighbourhood support was assessed by several questions on the perception of participant’s immediate neighbourhood including community support, service and amenities and satisfaction on the living area (more details on each original question via https://www.gov.uk/government/publications/community-life-survey-questionnaire-2013-to-2014). Potential covariates such as age, sex and ethnicity that could affect the carer profile were adjusted in the statistical modelling. Chi-square test and logistic regression modelling were performed to examine the variance in perception toward neighbourhoods between caregivers and non-caregivers. Venn diagrams were drawn to present the clusters between care provision and neighbourhood satisfaction. Effects were reported in odds ratios (OR) and 95% confidence intervals (CI), with *P* < 0.05 considered statistically significant. Statistical software STATA version 13.0 (STATA, College Station, TX, USA; more details via http://www.stata.com/) was used to perform all the statistical analyses.

## Results

### Neighbourhood support

Of 15,320 study participants, 2315 (16.0%) had a caring responsibility in England. People aged between 40 and 79 were more likely to provide caring than those aged under 40 (9.7%) or above 80 (9.4%), with a higher proportion in female (17.1%) than in male (14.8%). This included both professional caring in care homes and informal caring in the household. By ethnicity, people who classified as white (16.2%) or none (20.0%) were more likely to take up caring responsibility than Asian (14.8%), black (14.1%) or mixed (12.3%). Table 1 lists the associations between care provision and neighbourhood support. Clearly, there was not much variance in feeling belonging, comfortably asking neighbours to keep keys, comfortably asking neighbours to mind children, believing neighbours pulling together and trusting people in the neighbourhood between caregivers and non-caregivers. However, caregivers seemed to be more likely to chat to neighbours (OR 0.77, 95% CI 0.69–0.87, *P* < 0.001) and comfortably ask neighbours to help collect grocery than non-caregivers did (OR 0.89, 95% CI 0.81–0.98, *P* = 0.023).

**Table 1:** Associations between care provision and neighbourhood support

	No caregiving (*n* = 12,130, 84%)	Caregiving (*n* = 2315, 16.0%)	OR (95%CI)^a^	*P* value
Demographics				
Age				<0.001
16–39 year	3570 (90.3%)	382 (9.7%)	−	
40–59 years	4160 (79.9%)	1047 (20.1%)	−	
60–79 years	3509 (82.0%)	768 (18.0%)	−	
80+ years	656 (90.6%)	68 (9.4%)	−	
Sex				<0.001
Male	5683 (85.2%)	990 (14.8%)	−	
Female	6447 (83.0%)	1325 (17.1%)	−	
Ethnicity				0.202
Not classified	60 (80.0%)	15 (20.0%)	−	
White	10,899 (83.8%)	2108 (16.2%)	−	
Asian	616 (85.2%)	10 (14.8%)	−	
Black	292 (85.9%)	48 (14.1%)	−	
Mixed	263 (87.7%)	37 (12.3%)	−	
Perception toward neighbourhood				
Feeling belonging	7666 (83.3%)	1534 (16.7%)	0.92 (0.84–1.01)	0.087
No	4416 (85.1%)	774 (14.9%)	1.00	
Chatting to neighbours	9358 (83.1%)	1905 (16.9%)	0.77 (0.69–0.87)	<0.001
No	2756 (87.1%)	407 (12.9%)	1.00	
Comfortably asking neighbours to keep keys	840 (83.5%)	1692 (16.5%)	0.94 (0.85–1.04)	0.227
No	3537 (85.3%)	612 (14.8%)	1.00	
Comfortably asking neighbours to mind children	1360 (86.6%)	211 (13.4%)	1.09 (0.88–1.35)	0.438
No	1180 (86.5%)	184 (13.5%)	1.00	
Comfortably asking neighbours to collect grocery	7236 (83.0%)	1479 (17.0%)	0.89 (0.81–0.98)	0.023
No	4823 (85.5%)	820 (14.5%)	1.00	
Believing neighbours pulling together	6986 (83.7%)	1365 (16.4%)	1.02 (0.93–1.12)	0.676
No	4403 (83.9%)	848 (16.2%)	1.00	
Trusting people in the neighbourhood	9075 (83.8%)	1753 (16.2%)	1.02 (0.91–1.14)	0.716
No	2619 (84.4%)	484 (15.6%)	1.00	

^a^Adjusted for age, sex and ethnicity

### Neighbourhood services and amenities

There was also no difference in use or perception for a series of neighbourhood services or amenities including shops, pubs, parks, libraries, community centres, sports centres, youth clubs, health centres, chemists, post offices, primary and secondary schools, places of worship and public transportation (data not shown).

### Neighbourhood satisfaction

**Fig. 1:**
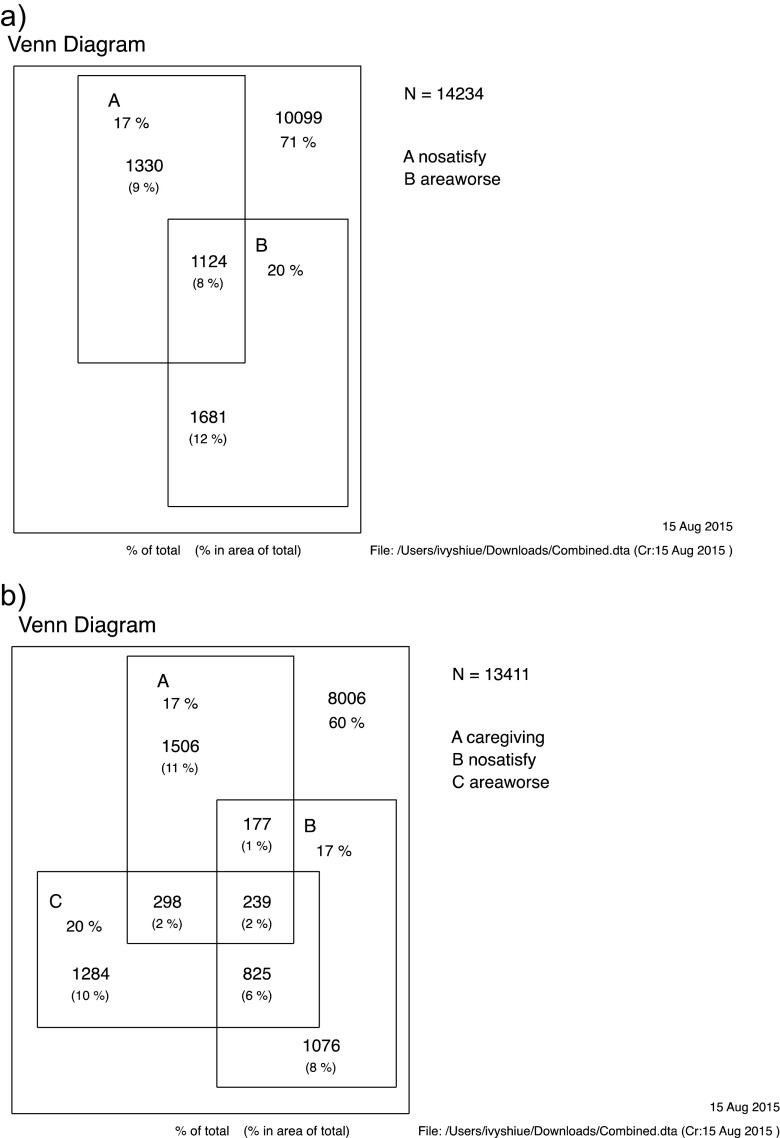
Distribution of care provision or not and neighbourhood satisfaction among the study participants

Figure 1 shows the clusters among caring provision, neighbourhood satisfaction and perception on its condition over the last 2 years. Apparently, nearly 30% of the study participants either had neighbourhood dissatisfaction or perceived their neighbourhoods have gone worse in the last 2 years while the likelihood was higher in caregivers (OR 1.17, 95% CI 1.05–1.32, *P* = 0.007 for dissatisfaction; OR 1.36, 95% CI 1.22–1.51, *P* < 0.001 for perceiving the neighbourhoods going worsened; also see Table 1). Such situation was more common in caregivers than non-caregivers. However, the specific reasons for these were unknown due to the lack of such data in the present survey.

## Discussion

It was not surprising to see more female than male to have been caregivers, whether professionally or informally. While the caregiving has been low paid or with no pay, it would be one of the sources in widening the social inequality due to the imbalanced spending on energy, time and pay. In the present study, there was not very much variance seen in neighbourhood support for both caregivers and non-caregivers. Instead, caregivers seemed to have a slightly greater social interaction than non-caregivers had through chatting to neighbours and kindly asking them to help collect grocery. Perhaps it is such process that has made them more aware of the change of neighbourhood than non-caregivers and therefore indicated their worry about the possible worsening condition over the last 2 years, whether real or perceived.

### Strengths and limitations

The present study has a few strengths. Firstly, the data were collected in a country-wide and population-based setting in the very recent years. Secondly, it was the first time to analyse the neighbourhood support for caregivers. However, there are still a few limitations that cannot be ignored. First, questions on neighbourhood support are not from a standard scale. This is because there has not been scientific research with a rigorous methodology to formulate. Therefore, there could be other missing aspects that were unable to be included. Second, how the neighbourhoods have gone worse was not included in the questionnaire. Therefore, the mechanism and pathway were unable to be disclosed and explained. Third, the causality cannot be established due to the cross-sectional study design in nature while the potential existing selection bias also means the research was not 100% representative through involving everyone to answer. However, it was assumed that the caring responsibilities have been constant until the household interview on neighbourhood support and perception. Taken together, future studies retaining the strengths and overcoming the limitations mentioned above with a longitudinal or qualitative approach across geographic regions to confirm such observations obtained in the present study would be warranted.

### Directions for future research, practice and policy

People whom had caring responsibilities seemed to have the similar level of neighbourhood support to others who had not caring responsibilities but tended to perceive their neighbourhoods unsatisfactory and worsened in the last 2 years. For future research, establishing a longitudinal neighbourhood monitoring surveillance for all people including patients, caregivers and healthy people altogether using a scientific approach could help with documenting evidence on the link between neighbourhoods and health, wellbeing and quality of life to guide any life satisfaction improvement. In addition, constructing a standard scale on neighbourhood support together with the local police force and social care and health services by regular examination through rigorous research would be suggested. For practice and policy, environmental health and nursing programs might need to extend education trainings and interventions to cover all neighbourhoods at different time points that can lessen both disease and caregiving burden and therefore to optimize health and quality of life.
